# First Phenol Carboxylation with CO_2_ on Carbon Nanostructured C@Fe-Al_2_O_3_ Hybrids in Aqueous Media under Mild Conditions

**DOI:** 10.3390/nano11010190

**Published:** 2021-01-13

**Authors:** Feliciano Calvo-Castañera, Jesús Álvarez-Rodríguez, Nuria Candela, Ángel Maroto-Valiente

**Affiliations:** 1 Dpto. de Química Inorgánica y Química Técnica, Facultad de Ciencias, UNED, Paseo Senda del Rey, 9, 28040 Madrid, Spain; fcalvo15@alumno.uned.es (F.C.-C.); amaroto@ccia.uned.es (Á.M.-V.); 2 Escuela Superior de Ingeniería y Tecnología, Universidad Internacional de La Rioja (UNIR), Avenida de la Paz, 137, 26006 Logroño, Spain; nuria.candela@unir.net

**Keywords:** carbon nanomaterials, alumina, hybrid, phenol conversion, CO_2_

## Abstract

Novel hybrid materials with integrated catalytic properties and hydrophobic response, C@Fe–Al_2_O_3_ hybrid samples, were presented and tested as catalysts for phenol reaction in aqueous solutions at atmospheric pressure and mild temperature conditions, using CO_2_ as a feedstock. A series of carbon-coated γ-alumina pellets (C@Fe–Al_2_O_3_) were synthesized and characterized by TGA, Brunauer–Emmett–Teller (BET) method, Raman spectroscopy, SEM, TEM, and XPS in order to get comprehensive knowledge of their properties at the nanoscale and relate them with their catalytic behavior. The results obtained correlated their catalytic activities with their carbon surface compositions. The application of these materials as active catalysts in the Kolbe–Schmitt reaction for CO_2_ conversion in aqueous media was proposed as an alternative reaction for the valorization of exhausts industrial effluents. In these early tests, the highest conversion of phenol was observed for the hybrid samples with the highest graphitic characteristic and the most hydrophobic behavior. Carboxylation products such as benzoic acid, p-hydroxybenzoic acid, and salicylic acid, have been identified under these experimental conditions.

## 1. Introduction

Most chemical reactions involving CO_2_ as a reagent are developed in the presence of highly reactive metal catalysts and high pressures and temperatures [1]. Carbon dioxide as a reactant is used to synthetize commodity chemicals as formic acid [2], methanol [3], light hydrocarbons [4,5,6], dimethyl ether [7], acetic acid [8], and cyclic organic carbonates from epoxides [9], alcohols [10,11], or oxetanes [12] as precursors of polycarbonates. Unsaturated compounds as dienes or allenes react with carbon dioxide to form lactones and carboxylic derivatives [13]. The reaction with ammonia leads to the urea formation, and in contact with other organic nitrogen-containing compounds as amines, aziridines, or propargyl amines, carbamic acids or oxazolidinones are produced [14].

Among them, one of the most classical reactions is the carboxylation of phenol, known as the Kolbe–Schmitt reaction, developed in 1860, when Kolbe obtained salicylic acid through electrophilic substitution by heating a phenol and sodium mixture with carbon dioxide at 80 atmospheres of pressure [15]. Several years after, Schmitt raised the pressure up to 130 atm to improve the salicylic acid yield [16]. This method has been the milestone for the aromatic hydroxy acid synthesis by carboxylation [17]. The Kolbe–Schmitt reaction is a well-known reaction for the industrial production of aromatic hydroxy acids, in which phenol carboxylation with CO_2_ is processed under high CO_2_ pressure (20–100 atm) and temperature (150–200 °C). However, the Kolbe–Schmitt advance in this reaction is inhibited by the presence of water, since dry phenoxide is considered as the determinant step. The selectivity of the Kolbe–Schmitt reaction is affected by temperature and pressure conditions and the type of alkaline metal used. On the other hand, the reaction carried out in aqueous conditions leads to different final molecules from those ones in the absence of water [18]. The Kolbe–Schmitt reaction is a universal process for global salicylic acid production, which is mainly demanded in the pharmaceutical industry to manufacture formulations such as acetylsalicylic acid, phenyl salicylate, sodium salicylate, methyl salicylate, and methyl salicylate. Salicylic acid at a commercial scale is produced by the Wacker process, based on the Kolbe–Schmitt reaction, for CO_2_ carboxylation of phenol catalyzed by dispersed dry sodium.

Nowadays, the CO_2_ emission is one of the greatest environmental problems for humans living on Earth and a challenge that the scientific community faces. Carbon dioxide reached in 2017 global emissions of 41.5 ± 4.4 billion tons of CO_2_ [19], of which 36.8 ± 2.0 Gt come from fossil fuels and industry [20]. However, around 130 million tons of carbon dioxide emissions are used as feedstocks, several orders of magnitude below the produced CO_2_. Only a few technologies use carbon dioxide as a feedstock for industrial applications, being the production of the most extended urea [21]. Many other compounds, such as alkylene carbonates, cyclic carbonates, polycarbonates, polyurethanes, and carboxylic acids, are found at a great distance according to their production tonnage [22]. The potential utilization of CO_2_ emitted by power plants represents a double advantage of the global warming mitigation and valorization of carbon dioxide as a reactant. 

With the intention of valuing CO_2_ emissions from postcombustion sources, innovative alternatives with industrial interest were proposed, not only to reduce the carbon footprint but also to take advantage of it as a resource. Therefore, the aim of this work is presenting initial results obtained with new hybrid hydrophobic carbonaceous materials (C@Fe–Al_2_O_3_) that display activity in the carboxylation reaction of phenol in an aqueous medium under mild conditions, with a CO_2_ gas stream under pressure and temperature conditions similar to those of a postcombustion process.

## 2. Materials and Methods 

### 2.1. Materials Preparation

Spherical γ-alumina pellets (3 mm diameter) were conditioned by drying at 383 K for 48 h, and then, iron was loaded by the wetness impregnation method using a diluted acetone solution of FeCl_3_·6H_2_O at room conditions [23]. C@Fe–Al_2_O_3_ hybrids were prepared by the carbon coating of iron-impregnated spheres (FeAl) via thermal catalytic vapor decomposition (CVD) in a 600 mm-length tubular quartz reactor with an inner diameter of 48 mm. Several lots of C@Fe–Al_2_O_3_ hybrids were synthetized at 723, 823, 923, 1023, and 1123 K with an acetylene flow for 30 min in the tubular reactor. Before the carbon source were fed, the impregnated spheres (FeAl) were activated by a thermal treatment in an atmosphere with a H_2_/N_2_ volume ratio of 1:1 (80 mL min^−1^) by an initial heating-up program of 10 K min^−1^ until 673 K and a stabilization step at this temperature for 60 min. Then, the reactor reached reaction temperature synthesis conditions with a final heating ramp of 10 K min^−1^. Afterwards, a flow of 40 mL min^−1^ of acetylene was mixed to the H_2_/N_2_ flow for 30 min. Once the reaction time was over, the as-synthesized C@Fe–Al_2_O_3_ hybrid was cooled down to room temperature (RT) under the inert gas flow.

### 2.2. Analytical Methods

The chemical analysis of the iron–impregnated samples (FeAl) was determined in homogeneously red-color bodies after disaggregation in boiling solutions with a HCl/Water volume ratio of 1:1 by the o-phenanthroline method (ASTM-E394) [24]. Briefly, the acid disaggregation of the samples, the reduction of Fe(III) dissolved to Fe(II) with hydroxylamine, and the iron phenanthroline complex were detected by UV-visible colorimetric analysis (Varian Cary 1).

Textural properties were determined by nitrogen adsorption–desorption isotherms at 77 K in a Micromeritics ASAP 2010 instrument. The surface area was calculated using the Brunauer–Emmett–Teller (S_BET_) method [25] based on the adsorption data in a relative pressure range of 0.05–0.20, and the pore size distributions were obtained from the desorption branch of the nitrogen isotherms by the Barrett–Joyner–Halenda (BJH) method.

Temperature-programmed TGA was carried out in a SDTQ600 5200 TA System. The sample with ca. 20 mg were placed in a holder while a dry air flow regulated with a mass flow controller at 100 mL min^−1^ was used during an thermal treatment at a speed of 2 K min^−1^ up to 1273 K.

SEM and TEM analyses were performed at a Hitachi S-3000N, FEI Nova NanoSEM 320 and JEOL JEM 2100 HT, respectively. Raman spectra were collected at RT with a Horiba Jobin Yvon T64000 spectrometer provided with a diode-pumped germanium solid-state detector, which operated at the liquid nitrogen temperature. An ion argon laser working at 514.532 nm was used as an excitation source with a power of ca. 200 mW. The samples were hold in a base and analyzed with a tangential incidence angle of 90° over a diffraction web of a 1.8 cm^−1^ resolution for an exposure time of 6 × 100 s. For spectral decomposition, Sadezky’s [26] method was followed using FITYK software [27], consistent in the separation of the spectrum in four different bands (G, D, D1, and D2) with a Lorentzian shape and one (D3) with a Gaussian shape due to the statistical distribution of the amorphous carbon in the interstitial of graphitic layers. The size of the graphitic coherent domains perpendicular to the c-axis (La) were determined through the Cançado equation [28] derived for the Tuinstra and Koenig relationship [29] regarding a 514.5 nm laser. On the other hand, the relative intensity of the D3 band (I_D3_/I_G_) [30] was determined to verify the proportion of amorphous carbon in the samples.

The XPS spectra were recorded with an Omicron spectrometer equipped with an EA-125 hemispherical electron multichannel analyzer and an unmonochromatized Mg Kα X-ray source at 100 W and a pass energy of 50 eV. The samples were mounted on a copper holder and introduced into the chamber and degassed in dynamic vacuum below 10^−8^ Pa prior to analysis. The registered spectra data were analyzed using CasaXPS software [31]. The wettability of the samples was determined as the interfacial solid–liquid contact angle of the spherical hybrids on ultrapure Millipore water. The samples were placed in the middle of a bath stabilized at 25 °C, and their contact angles were measured as a result of the hydrophobicity of their surfaces.

### 2.3. Phenol Conversion

The study of the C@Fe–Al_2_O_3_ hybrid behavior as a catalyst was carried out in a 250 mL three-necked round-bottom flask reactor fitted with a condenser and a bubbling catheter. Experiments were developed at an atmospheric pressure and a constant reflux temperature, with an initial feed of 100 mL of phenol aqueous solutions (20 mmol L^−1^) added into the glass reactor. A glass catheter bubbled a continuous 40 mL min^−1^ CO_2_ stream (99.999%; Nippon Gases). Initially, the system was purged with carbon dioxide for 30 min and subsequently heated to a reflux temperature of 370 K. Afterwards, every run was initiated by adding 200 mg of the fresh catalyst, whereas 2 mL samples were withdrawn at predetermined timed intervals. A variable stirring speed was kept to avoid the friction and erosion between the catalyst hybrid spheres, as well as to avoid contact with the walls of the reactor. The concentration of phenol was quantified using an Thermo Electron High Performance Liquid Chromatography (HPLC) equipped with a Agilent ZORBAX Eclipse Plus C18 reverse-phase Column (4.6 mm × 150 mm, 5 μm) in an isocratic eluent-water(H_3_PO_4_)/acetonitrile mixture (*v*/*v:* 80/20) pumped at a flow rate of 1.0 mL min^−1^. Detection was performed with a UV-visible spectrophotometer (Thermo Electron) at 270 nm. In these conditions, the phenol retention time observed was 7.8 min.

Conversion was calculated as the ratio of the moles of phenol converted amount to the initial amount fed. Selectivity was defined as moles of phenol transformed towards the main reaction products (C6 and C7) per 100 moles of phenol converted into quantified products.

## 3. Results

### 3.1. Characterization of the Hybrid Precursors

**Figure 1:**
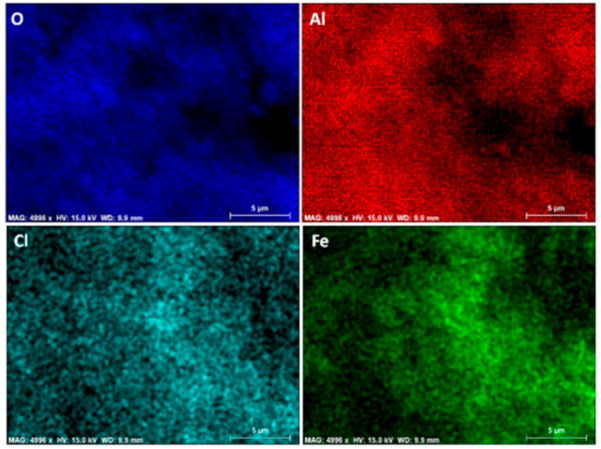
Mapping of the elemental distributions in sample FeAl (oxygen, aluminum, chloride, and iron) by Energy Dispersive X-ray spectroscopy (EDX) chemical analyses.

**Figure 2:**
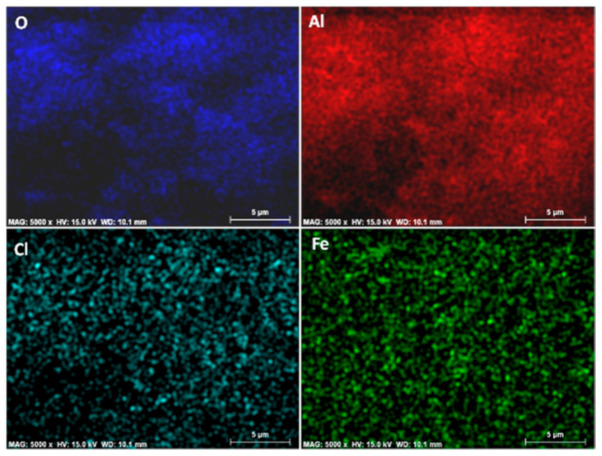
Mapping of the elemental distributions in sample FeAlR-1123 (oxygen, aluminum, chloride, and iron) by EDX chemical analyses.

The chemical analyses of iron content from the impregnated spheres (FeAl) were carried out. It reached a 2.48 wt%, showing a homogenous iron distribution on the spheres, as evidences in Figure 1. This amount was kept in the hybrid precursors (FeAlR) along the thermal synthetic program, consisting of reduction steps and heating ramps, until the temperature of the hybrid synthesis was homogenous, as evidences in Figure 2.

The surface area (S_BET_), pore volume, and pore characteristics of the impregnated (FeAl) and reduced spheres (FeAlR) are summarized in Table 1. These values of γ-alumina are consistent with previously reported values [32]. For all samples, type IV nitrogen adsorption isotherms were observed, with a type H3 hysteresis loop in IUPAC classification, which suggested the presence of mesopores in all of the samples. It is commonly associated with disordered pore textural structures found on solids with a very wide distribution of pore size, as lamellar or slit pores. Although the pore volume and surface area were reduced with the increase of the synthetic temperature of the hybrids, their pore distribution was close to 5 nm.

### 3.2. Characterization of the C@Fe–Al_2_O_3_ Hybrids

The surfaces of the C@Fe–Al_2_O_3_ hybrid samples showed a dark black coating after thermal CVD, without observable external heterogeneous regions, while a middle cross-sectional area revealed a black egg-shell radial distribution over a white core of γ-alumina (Figure S1). All the hybrid samples showed mechanical stability except the sample prepared at 1123 K. This fact could be explained due to a change in the grain size of the support due to the transition of the alumina to δ-alumina caused by increasing temperature [32,33,34] or due to the fragmentation of the support during carbon growth.

Type IV nitrogen adsorption isotherms were observed for the C@Fe–Al_2_O_3_ hybrids prepared at 723, 823, 923, and 1023 K, with type H3 hysteresis in IUPAC classification, which indicated that mesopores were present in all of samples. Moreover, these results showed that the main textural contribution was like the hybrid precursors. The surface area analyses by the BET method resulted in lower surface areas for these samples than for the γ-Al_2_O_3_ pellet. Additionally, it was established that higher surface area values were obtained with the increase of coating reaction temperatures (Table 2).

**Figure 3:**
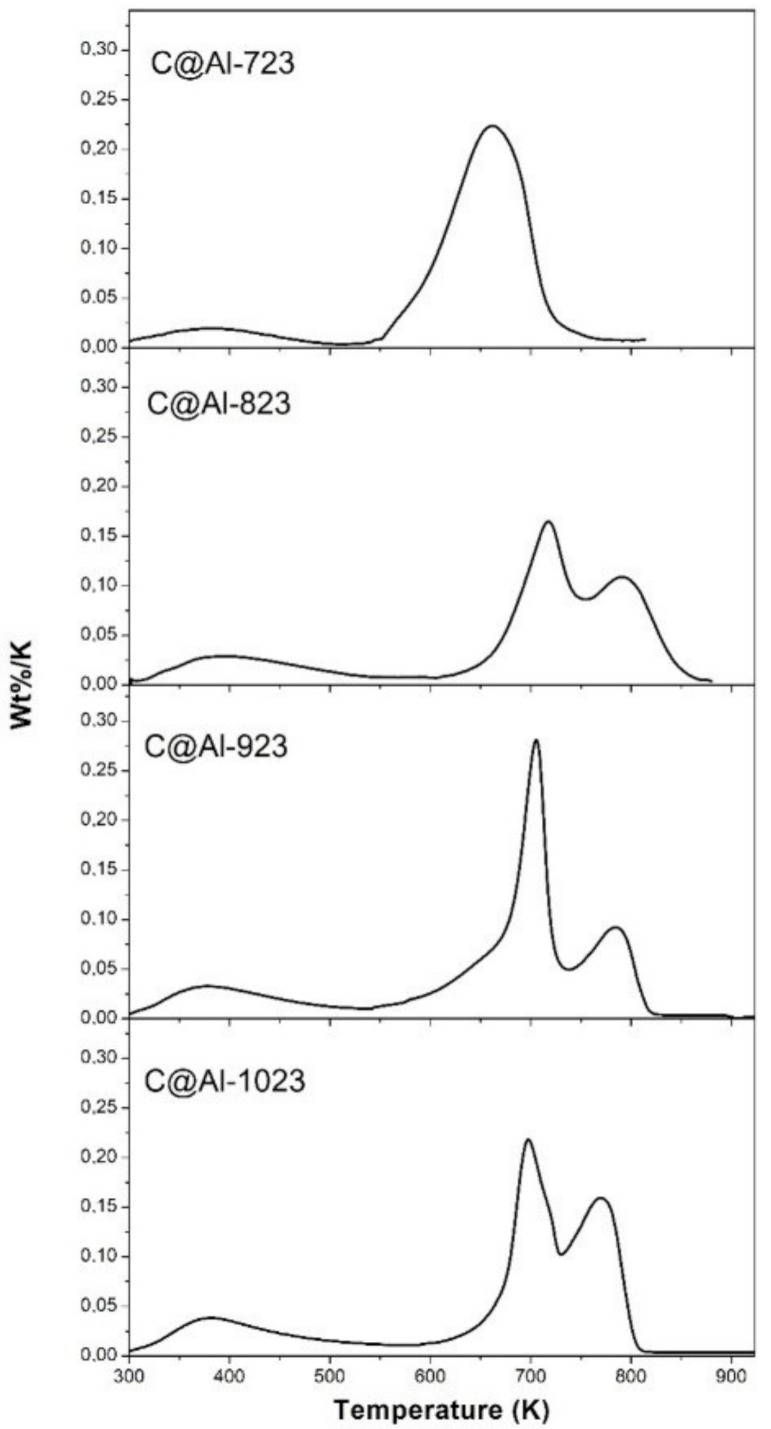
Profiles of the differential TGA (DTGA) for the thermal stability of the C@Fe–Al_2_O_3_ hybrids in air.

The TGA of the C@Fe–Al_2_O_3_ hybrids in air (Table 2) showed the yield of carbon synthetic growth was larger at lower reaction temperatures for the preparation of the C@Fe–Al_2_O_3_ hybrids. The differential TGA (DTGA) of the carbon-coated samples showed two main peaks (Figure 3), the former with a smooth slope at lower temperatures around 400 K related to the water separation and the latter with a higher slope above 700 K attributed to two contributions for samples C@Al-823, C@Al-923, and C@Al-1023. A study of derivative weight signal (Table 2) could a bimodal population of carbon species with combustion in synthetic air around 700–740 K (T1) related to amorphous carbon and the main peaks above 810–820 K (T2) assigned to more stable carbonaceous nanostructures as carbon nanotubes (CNTs) or carbon nanofibers (CNFs). These results are similar to previous reports of carbon nanomaterial studies [23,32,33]. On the other hand, the FWHM results gave an idea about the homogeneity of each subpart. In this case, there was a similarity for the more nanostructured carbon species (T2) for every hybrid samples analyzed; however, an outstanding homogeneity for the amorphous phase (T1) was observed in C@Al-923.

**Figure 4:**
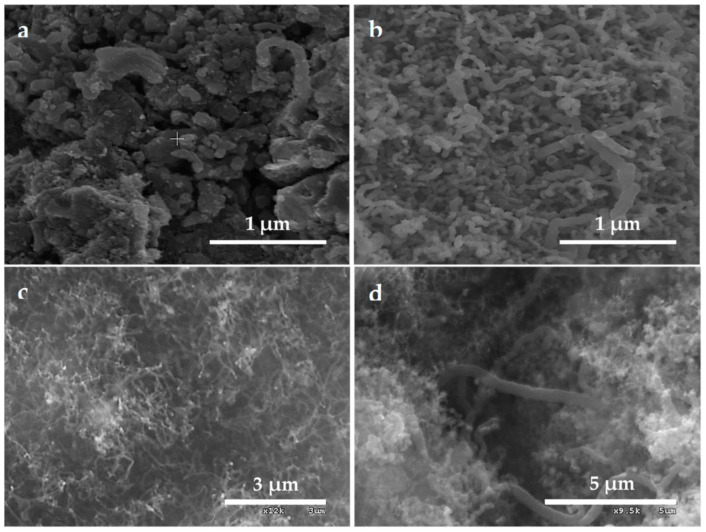
SEM images of hybrid samples: (**a**) C@Al-723; (**b**) C@Al-823; (**c**) C@Al-923; (**d**) C@Al-1123.

The SEM micrographs confirmed that carbonaceous coverage was completed after the synthetic process, with the appearance of carbonaceous nanofilaments for hybrids prepared above 823 K (Figure 4) with the habit of the nanowhisker-like growth. The higher the operation temperature, the higher extensive surface carbon filamentous and the related shapes coating were detected. In the same way, the cross-sectional profile of the C@Fe–Al_2_O_3_ hybrid spheres showed as much carbon yield (T1) as deeper internal layers were occupied, while the nanofilamentous species that could be considered as carbon nanotubes or carbon nanofibers (T2) were grown on the surface of the γ-Al_2_O_3_ spheres. Attending to the diameter and length of the nanofibers, it was possible to differentiate several groups for each temperature of operation. Nanofibers were shown on the surface of the hybrids in sample C@Al-723 (Figure S2), while larger length nanofibers were obtained in C@Al-1023 and C@Al-923 (2–10 μm) than in C@Al-823 (1–2 μm) In a parallel way, the higher the synthetic temperature, the larger the nanofiber diameters were grown. Therefore, groups of nanofibers were observed in micrographs with length of 25 nm, 45–60 nm, and 120 nm in sample C@Al-823 (Figure S3), 35 nm, 50–70 nm, and 100–120 nm in sample C@Al-923 (Figure S4), and 50 nm and 200–300 nm in sample C@Al-1023 (Figure S5).

The Raman spectroscopy results provided the information of structural characteristics of carbon growth on hybrid C@Fe-Al_2_O_3_ samples (Figure S6). Moreover, the FeAl sample spectra showed a large amount of different intensity peaks throughout its wavenumber range, without the growth bands being appreciated as they appeared in the samples where carbon fixation was made. The C@Fe–Al_2_O_3_ samples synthetized at 723, 823, 923, and 1023 K showed a characteristic signal of species with ordered graphitic carbon structures in the range of 1000–2000 cm^−1^ (Table 3).

First, the D band [35] in plane carbon ring breathing mode (A1g mode), which is related to structural defects associated with the degree of disorder in the graphitic structure or the greater appearance of edges or discontinuities in the graphite sheet, was detected. On the other hand, the G band [36] that indicated vibrational sp^2^-bonded modes of carbon atoms within aromatic carbon rings and the planar displacements on the graphite sheets was also found. The deconvolution of the spectra showed the D_1_ and D_2_ bands linked to the interstitial defects between the layers and amorphous carbon presence in nanostructured carbons as graphene oxide [37], CNFs [38], and CNTs [39,40]. On the other hand, in all the species studied, a shift in the baseline and the intermediate zone between the D and G bands was observed, which should be related to the overlapping of spectral peaks produced by the presence of unstructured or amorphous graphitic carbon [41,42] with sp^3^ hybridization. 

Outside the frequency range of 900–1900 cm^−1^, no other significant bands were observed. The results from the analyses of the position, width and intensity of the D and G bands are shown in Table 3. The main D band appeared in the frequency interval between 1338 and 1343 cm^−1^, and the width of this band decreased from 161 to 130 cm^−1^ when the operating temperature increased, which indicated an increase in the order of the crystal structure or a reduction in the number of boundary edges in the crystals due to the greater continuity and size of these crystals. The decrease of the values when increasing the preparation temperature also occurred with the D_1_ band, appeared between 1611 and 1612 cm^−1^, and related to the disorder in the graphitic structure or a greater number of edges or discontinuities of sheet [35]. The G band appeared in the frequency range of 1588–1594 cm^−1^, keeping the width of the band in similar values, without a clear tendency depending on the operating temperature. The planar microcrystalline size (L_a_) given by the I_D_/I_G_ ratio and determined by the ratio of the areas of the bands, showed a growth of the crystalline dimensions parallel to the operating temperature, varying from 5.19 nm for the obtained carbonaceous species at 723 K and to 8.11 nm for those obtained at 1023 K. This fact is in line with the decrease in the bandwidth of the D band, which corroborated a higher crystallinity and a lower number of defects in hybrid samples prepared at higher operating temperatures.

In the Raman spectra, the D_2_ band appeared between 1180 and 1233 cm^−1^, especially for the samples prepared at lower temperatures, of which the widths were 98 and 89 cm^−1^ for samples C@Al-723 and C@Al-823, respectively. Otherwise, in those synthesized samples at 923 and 1023 K, their presence was obtained by the curves convolution. This band was due to poorly organized carbonaceous species with sp^2^–sp^3^ bonds or the vibrations of the C–C or C=C bonds in polyene structures. In parallel, the band appearing in the range of 1500–1508 cm^−1^ was also associated with the presence of carbon sp^3^ hybridization or interstitial defects between layers, so the decrease in the I_D3_/I_G_ ratio indicated a lower content of amorphous carbon, which was more pronounced in the species obtained at a higher synthetic temperature. On the other hand, the absence of CNFs, verified by SEM, and the higher I_D3_/I_G_ ratio observed in C@Al-723 could be compatible with the presence of “glassy carbon”-type structures as acetylene black.

**Figure 5:**
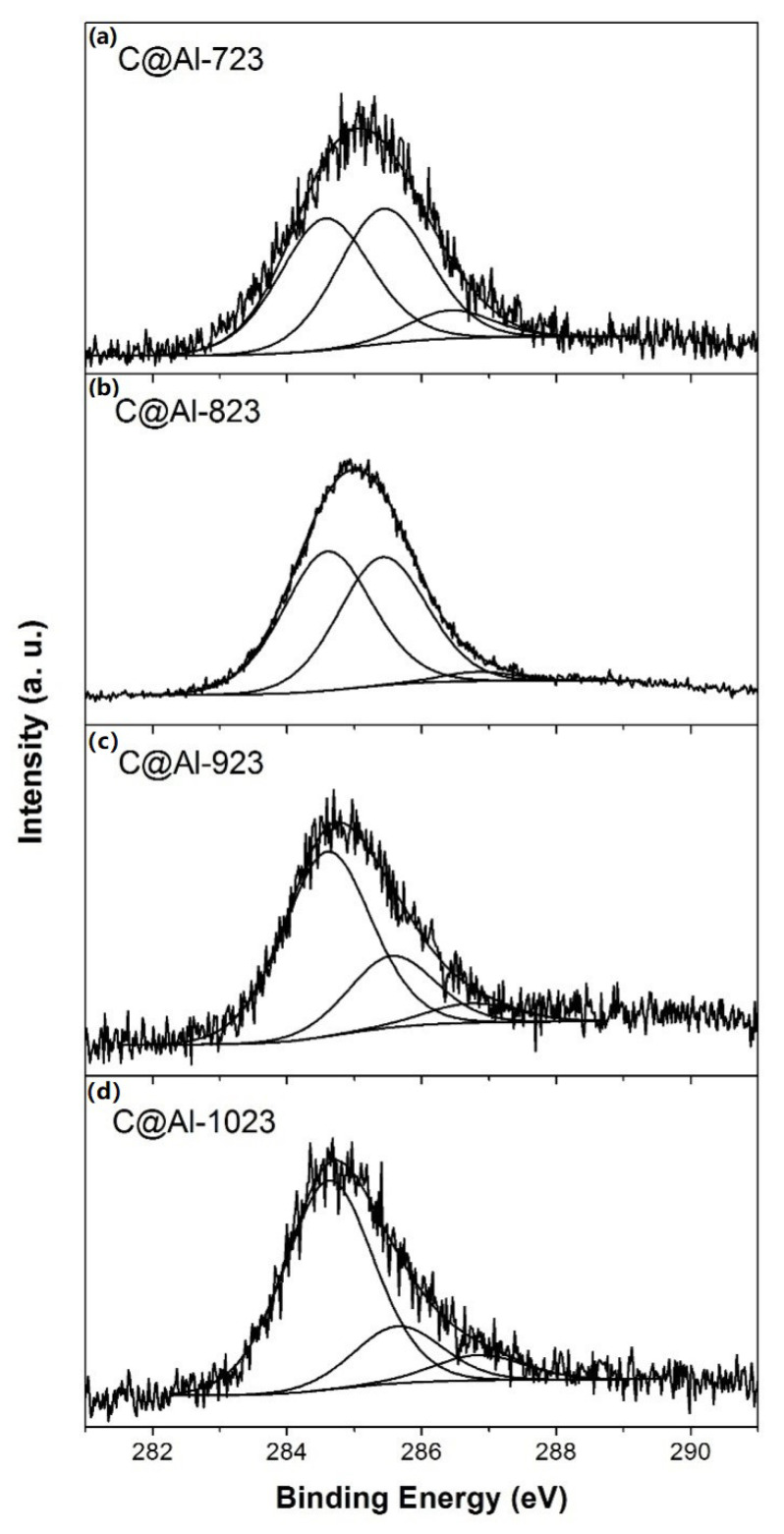
XPS spectra in region C_1s_ of different samples: (**a**) C@Al-723; (**b**) C@Al-823; (**c**) C@Al-923; (**d**) C@Al-1023.

The XPS spectra of the hybrid samples showed only two contributions from two main peaks due to the C1s and O1s species. The deconvolution of these peaks showed different contributions of C–C(sp^2^), C–C(sp^3^), and –C–O–C–(ether) previously reported [43,44,45,46,47]. The C1s region was fitted with three different peaks (Figure 5) [23,47]: one peak due to the graphitic carbon in polyaromatic structures (C=C: ∼284.6 eV), one peak attributed to the formation of aliphatic carbon (C–C: ∼285.4 eV), and one peak for carbon atoms bound to oxygen (C–O: ∼286.8 eV). Table 4 shows the percentages for the different species in the C1s region and the O/C atomic ratio. The increment in synthesis temperature enhanced the relative amount the of sp^2^/sp^3^ ratio that can be related to a higher graphitic character.

**Figure 6:**
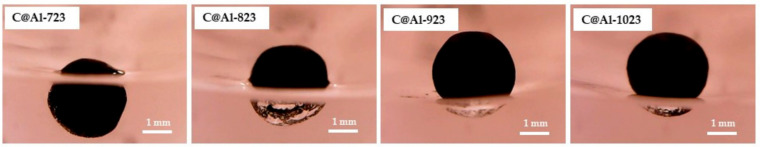
Images of the C@Fe–Al_2_O_3_ hybrid samples floating at the distilled water surface.

The measurements of the three-phase contact angle at the air–water interface were employed as a reference for the hydrophobicity analysis of this kind of spherical C@Fe–Al_2_O_3_ hybrids [48,49], of which the floatability properties were influenced by the temperature of synthesis as shown in Figure 6. The contact angles obtained were 110° on C@Al-823, 122° on C@Al-923, and 126° on C@Al-1023. The contact angle values of >90° confirmed that the samples synthesized at temperatures of ≥823 K showed a hydrophobic behavior as previously reported C@Fe–Al_2_O_3_ hybrids [23]. In contrast, the contact angle measured on C@Al_2_O_3_-723 (74°) indicated the sample was nonhydrophobic. 

**Figure 7:**
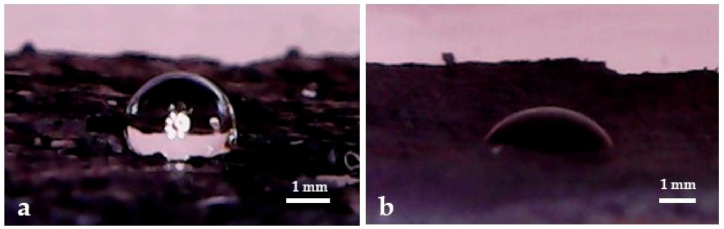
Images of the droplets water over MWCNTs (**a**) and carbon nanofibers (CNFs) (**b**).

The hydrophobicity of some carbon materials is well-known, which should be taken as an advantage in some applications. However, these materials have not been reported as superhydrophobic materials yet. In fact, surface groups, commonly produced in the synthesis of these materials, made their behavior far from hydrophobicity. In the case of CNFs and of Multiwall carbon nanotubes (MWCNTs) (Figure 7), the measured water contact angles on the surface were 60° and 120°, respectively, far from superhydrophobic behavior [50,51]. Therefore, the origin of the higher hydrophobicity of this kind of spherical C@Fe–Al_2_O_3_ hybrids would be related to the external carbon nanowhiskers growth, of which the distributions in the surface of the spherical samples evidenced by TGA, SEM, TEM and Raman spectroscopy were consistent with a gecko effect. This property should be fundamental to the application of these hybrids in aqueous solutions in the catalytic test.

### 3.3. Phenol Conversion in C@Fe–Al_2_O_3_ Hybrids

**Figure 8:**
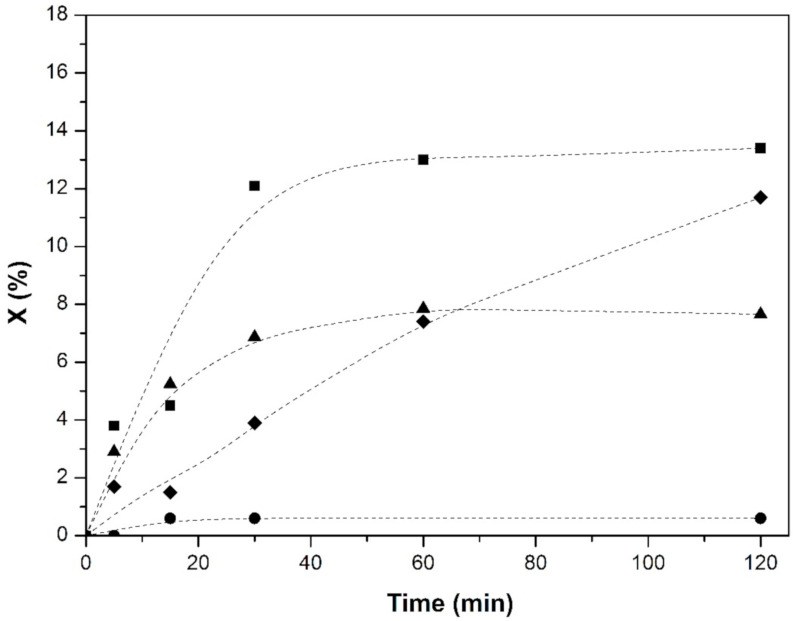
Reaction profiles of the C@Fe–Al_2_O_3_ hybrid samples: (●) C@Al-723, (◆) C@Al-823, (■) C@Al-923, and (▲) C@Al-1023.

The reaction tests for the C@Fe–Al_2_O_3_ hybrid samples on the phenol conversion in aqueous solutions at 370 K and ambient pressure with a 40 mL min^−1^ flow of CO_2_ were conducted in batch mode. C@Al-723, C@Al-823, C@Al-923, and C@Al-1123 were active as catalysts in phenol conversion (Figure 8). In this sense, the hybrids wettability properties showed, as their structural formulation, that they could be necessary in order to observe phenol conversion, as can be evidenced by the fact that no reaction was observed under the same reaction conditions with γ-alumina spheres or FeAl spheres. Although values were far from full conversion, it is in the same range of those reported in the most common reaction, converting CO_2_ to methanol in heterogeneous catalysis [52,53]. The porous distribution of the samples allowed for the phenol and CO_2_ spread in the hybrids to the active sites by diffusion, while the aqueous solution was maintained outside the spheres due to their hydrophobicity properties created by the nanofibers and nanowhiskers-like growth, resulting in a biphasic media. 

The reaction tests in aqueous media in batch mode under different gas flows of N_2_, air, or CO_2_ only resulted in phenol conversion under the carbon dioxide flow. As stated in the introduction, this finding has never been reported in these conditions in the literature. In fact, how this process was typically carried out at 440 K and 10 MPa [16] and the inhibition effect in the presence of water of the Kolbe–Schmitt carboxylation (Figure S7) [54,55,56] by blocking the cation-induced electrophilic replacement in the aromatic ring [57,58] were previously reported. Thus, it is an evidence that phenol conversion is possible due to the characteristics of these hybrid materials as their hydrophobicity (in order to reduce the inhibition effect of water) and surface composition (carbon coating, Fe species, or a mixed site of both should take place as active sites).

**Figure 9:**
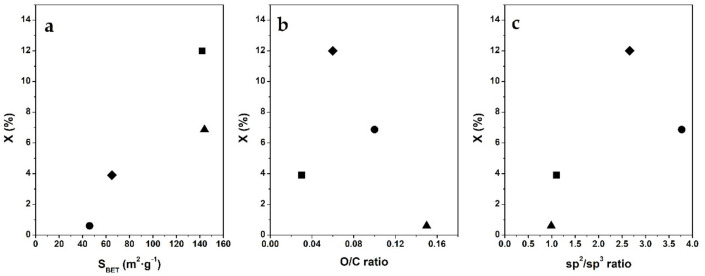
Correlations of the C@Fe–Al_2_O_3_ hybrid samples: (●) C@Al-723, (◆) C@Al-823, (■) C@Al-923, and (▲) C@Al-1023. (**a**) textural properties (as specific surface area, S_BET_); (**b**) oxygenated surface groups (as XPS O/C ratio); and (**c**) carbon-type (as XPS sp^2^/sp^3^ ratio) of carbon materials in hybrids with phenol conversion at a 30 min reaction.

The catalytic behaviors of these sample in the phenol conversion showed different shape profiles for all of them with an initial conversion slope of the curves in the order of C@Al-923 > C@Al-1023 > C@Al-823 >> C@Al-723 and reached up at 120 min in the order of C@Al-923 > C@Al-823 > C@Al-1023 >> C@Al-723. The calculated rate constant for phenol conversion was 0.02 min^–1^ with C@Al-723, 0.13 min^−1^ with C@Al-823, 0.2 min^−1^ with C@Al-923, and 0.4 min^−1^ with C@Al-1023. Therefore, differences in carbon morphology should explain these differences in phenol reaction conversion. In this sense, the acetylene carbon structure, proposed in sample C@Al-723, with higher I_D3_/I_G_ and O/C ratios, was less active in the reaction. On the other hand, similar I_D3_/I_G_ ratios but different catalytic activities, specific surfaces (S_BET_), and O/C ratios were observed in samples C@Al-823, C@Al-923, and C@Al-1023. Moreover, the dependences of the conversion on those variables were studied in order to get more insights about this hybrid’s phenol conversion and specific surface (Figure 9a, S_BET_), carbonaceous borders (Figure 9b, O/C ratio), and graphitic carbonaceous structure (Figure 9c, sp^3^/sp^2^ ratio). 

Therefore, a positive correlation between the surface area of hybrids and the conversion was detected (Figure 9a), since greater S_BET_ materials displayed higher phenol conversion. Attending to the order of C@Al-1023 > C@Al-923 > C@Al-823 > C@Al-723 in the reduction of specific surface with the reduction of phenol conversion, as the pore volumes were similar in the four samples (Table 1), this behavior could be related to the pore size which should be related to the carbon nanostructures grown during the hybrids preparation (in the external volume of the spheres; Figures S1–S6). Hence, as this conversion increased in opposition to the carbon contents of the samples (Table 2), this effect could be related to the carbon nanostructures. 

In this sense, among the main characteristics of carbon nanostructures were the extension of basal planes (sp^2^ carbon bonds) and surface oxygenated groups (O/C ratio). A negative correlation between the O/C ratio (Figure 9b) and the conversion was found, and the higher the surface oxygenated groups of the carbon materials, the lower the phenol conversion, while a positive effect of sp^2^ carbon bonds is shown (Figure 9c). Therefore, the reaction took place in the outer carbon coverage of the spheres, probably in the site with sp^2^ carbon (as the basal planes of nanofibers) but the Fe participation was not resolved.

At this time, it was not possible to elucidate a mechanism, but throughout the time of phenol reaction, the C7 compounds (benzoic acid, p-hydroxybenzoic acid, and salicylic acid) and the C6 compounds (benzoquinone, hydroquinone, and catechol) were identified, with a relatively low total yield of <5%, measure by the HPLC technique, in the same range of other authors’ findings to CO_2_ hydrogenation [56]. The selectivities of benzoquinone, hydroquinone, catechol, and acids of low carbon chains among the main products measured for all the samples were 59–70%, 5–10%, 17–23%, and <5%, respectively, which should be related with phenol degradation via an oxidation process [23]. Neither double carboxylation nor meta-carboxylation product was detected. The formation of benzoic acid (0–6%), p-hydroxybenzoic (<1%) acid, and salicylic acid (0–10%), which presented an additional carbon in the molecular structure, should be related with the formation of C–C bonds by carboxylation [53,54,55]. The C@Al-923 and C@Al-1023 hybrids showed the highest relative amount of salicylic acid among carboxylation products (>90% of C7 compounds). p-hydroxybenzoic acid was the unique C7 carboxylation product detected for sample C@Al-823. Finally, similar amounts of salicylic and benzoic acid were observed on the C@Al-723 hybrids. The experiments conducted with different gases dissolved in aqueous bath media (N_2_, air, CO_2_, and CO_2_/air) only confirmed the presence of C7 compounds under carbon-dioxide flow, and consequently, carboxylation reaction was confirmed. Therefore, a competitive reaction pathway of phenol oxidation reaction (C6 compounds) and carboxylation reaction (C7 compounds) should be studied more in detail, but this is out of the scope of this work.

Elsewhere, these findings—the detection of C7 compounds in phenol conversion on the C@Fe–Al_2_O_3_ hybrids under mild conditions in aqueous media—confirmed that the main target of this work as an early approximation for an alternative process to uptake and the valorization of CO_2_ by carboxylation of phenol has been achieved.

## 4. Conclusions

C@Fe–Al_2_O_3_ hybrid materials have been synthetized and analyzed as catalysts for the carboxylation of phenol in an aqueous solution with CO_2_ under mild conditions of pressure and temperature. All the synthesized hybrid spheres have presented an extensive dark black coating after CVD. The textural surface analysis revealed all carbon-coated samples obtained reductions in surface and pore volume, in comparison to the γ-alumina support.

The TGA has shown how the synthetic temperature of the hybrids resulted in two groups, which were those synthetized at 723 K and those to 823 K, 923 K and 1023 K. The SEM images of the hybrids confirmed the full coating of the sphere with a carbonaceous nanomaterial and how hybrids prepared at higher synthetic temperatures showed nanofilamentous forms with graphitic structures that were compatible with CNFs. The Raman spectra showed that the less crystalline hybrids (C@Al-723) corresponded to glassy carbon (acetylene black). The XPS analysis demonstrated that a higher temperature led to a greater graphitic characteristic for carbon growth, which was confirmed by the three-phase contact angle obtained at the air–water interface hydrophobic behavior.

Phenol conversion in the reaction with CO_2_ was higher using higher-surface-area, more graphitic (O/C ratio of 0.08 and a sp^2^/sp^3^ ratio of 2.8) and hydrophobic synthesized samples (C@Al-923 and C@Al-1023). The analysis of reaction products suggested a competitive complex pathway, which included products obtained from oxidation and carboxylation reactions. The oxidation of phenol to benzoquinone, hydroquinone, or catechol occurred, while the carboxylation of phenols to benzoic acid, p-hydroxybenzoic acid, and salicylic acid are detected.

## Figures and Tables

**Table 1:** Textural properties of γ-Al2O3 spheres and pre-synthetic hybrid samples.

Sample	Average Pore Size ^1^ (nm)	Pore Volume ^1^ (cm^3^ g^−1^)	S_BET_ (cm^3^ g^−1^)
γ-Al_2_O_3_	4.6	0.42	293
FeAl	5.0	0.40	247
FeAlR-723	4.8	0.38	255
FeAlR-823	4.7	0.40	255
FeAlR-923	5.0	0.39	221
FeAlR-1023	5.7	0.41	193
FeAlR-1123	7.3	0.42	155

^1^ Determined by the Barrett–Joyner–Halenda (BJH) method for the desorption curve.

**Table 2:** Textural properties and thermal stability of the C@Fe–Al_2_O_3_ hybrids in air.

Sample	S_BET_ (cm^3^ g^−1^)	Carbon ^1^ (wt%)	T1 (K)	Carbon (wt%)	FWHM (K)	T2 (K)	Carbon (wt%)	FWHM (K)
C@Al-723	46	22.4	692	100	85.7	-	-	-
C@Al-823	66	19.4	745	64.2	59.7	817	35.8	60.0
C@Al-923	142	18.0	731	64.3	29.1	811	35.7	57.8
C@Al-1023	145	12.6	721	54.7	60.9	809	45.3	56.2

^1^ Total weight loss (by TGA).

**Table 3:** Main data for the Raman spectra of the hybrid samples.

Hybrid Sample	D_1_^1^		D_2_		D_3_		D		G ^2^		I_D3_/I_G_	L_a_^3^ nm
	cm^−1^	FWHM	cm^−1^	FWHM	cm^−1^	FWHM	cm^−1^	FWHM	cm^−1^	FWHM		
C@Al-723	1233	98	1611	42	1500	174	1343	161	1588	50	0.62	5.19
C@Al-823	1180	89	1611	40	1508	159	1341	161	1589	66	0.38	7.24
C@Al-923	1197	100	1611	31	1508	144	1338	127	1594	56	0.38	7.58
C@Al-1023	1200	112	1612	21	1504	142	1342	130	1589	62	0.28	8.11

^1^ Interstitial defects between layers (D_1_). ^2^ Graphite band due to the symmetry of the hexagonal lattice (G). ^3^ Planar microcrystalline size (L_a_).

**Table 4:** Summary of XPS spectra in region C1s for C@Fe–Al_2_O_3_ hybrid samples.

Hybrid		C1s (wt%)		sp^2^/sp^3^	O/C
Sample	C=C	C–C	C–O	Ratio	Ratio
C@Al-723	41.40	44.53	14.07	0.99	0.15
C@Al-823	44.41	52.41	3.18	1.10	0.03
C@Al-923	68.08	26.25	5.68	2.66	0.06
C@Al-1023	72.11	19.11	8.78	3.77	0.10

## Supplementary Materials

The following are available online at https://www.mdpi.com/2079-4991/11/1/190/s1, Figure S1: Cross-section images of hybrid samples: (a) C@Al-723; (b) C@Al-823; (c) C@Al-923; (d) C@Al-1123., Figure S2: SEM images of C@Al-723 hybrid sample, Figure S3: SEM images of C@Al-823 hybrid sample, Figure S4: SEM images of C@Al-923 hybrid sample, Figure S5: SEM images of C@Al-1023 hybrid sample, Figure S6. Raman spectra of impregnated sphere (FeAl) and C@Fe-Al2O3 hybrids, Figure S7. Possible products obtained from direct carboxylation of phenol.
