# Supplementary material for: First Phenol Carboxylation with CO2 on Carbon Nanostructured C@Fe-Al2O3 Hybrids in Aqueous Media under Mild Conditions
Source: Nanomaterials (Basel). 2021 Jan 13;11(1):190. doi: 10.3390/nano11010190 (PMC7828619; doi:10.3390/nano11010190)
Supplement: Supplementary file 1 [file nanomaterials-11-00190-s001.pdf]

Supplement Information

First phenol carboxylation with CO_2 on carbon nanostructured C@Fe-Al_2O_3 hybrids in aqueous media under mild conditions

Feliciano Calvo-Castañera [1], Jesús Álvarez-Rodríguez [1,*], Nuria Candela [2] and Ángel Maroto-Valiente [1]

[1] Dpto. de Química Inorgánica y Química Técnica, Facultad de Ciencias, UNED, Paseo Senda del Rey, 9, 28040 Madrid.

[2] Escuela Superior de Ingeniería y Tecnología, Universidad Internacional de La Rioja (UNIR), Avenida de la Paz, 137, 26006 Logroño, La Rioja, Spain.

* Correspondence: jalvarez@ccia.uned.es

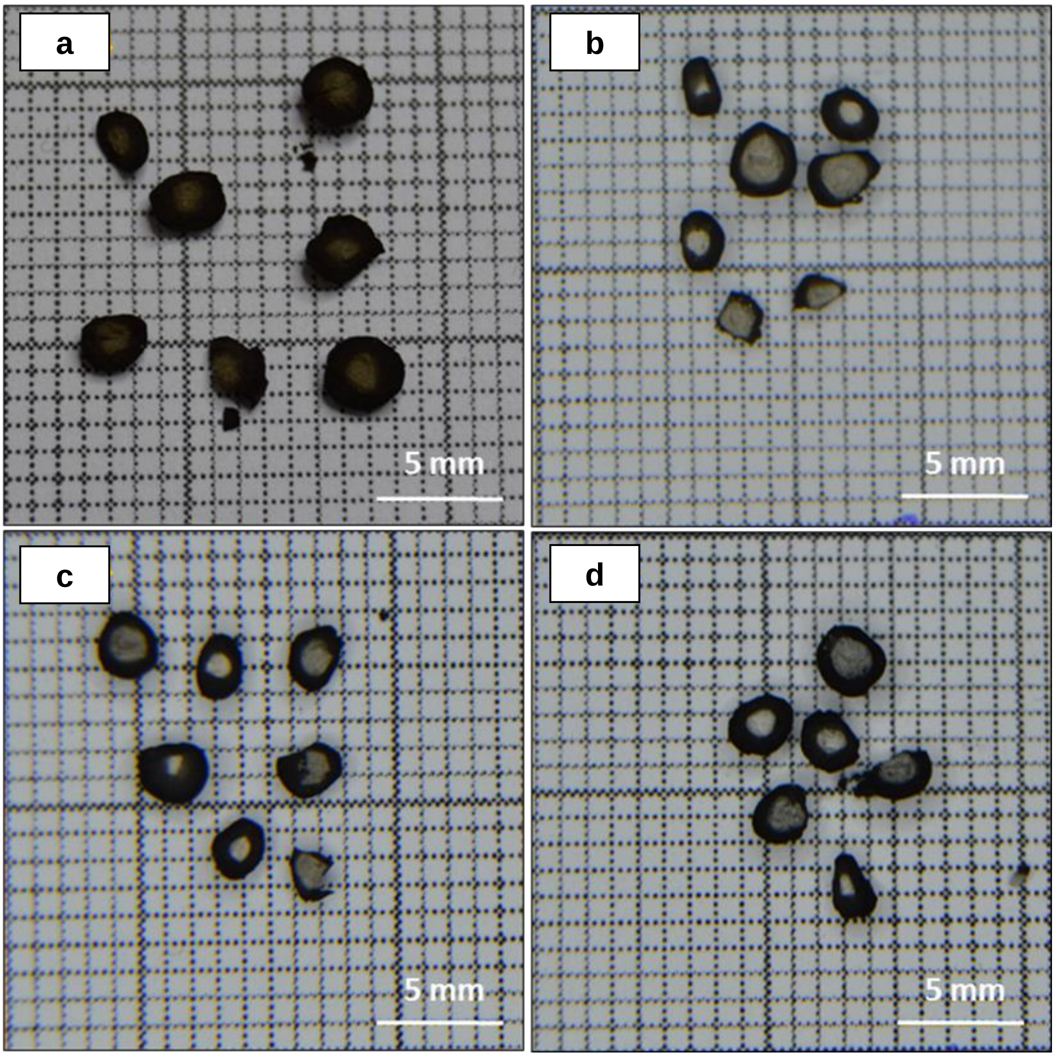

Figure S1. Cross-section images of hybrid samples: (**a**) C@Al-723; (**b**) C@Al-823; (**c**) C@Al-923; (**d**) C@Al-1123.

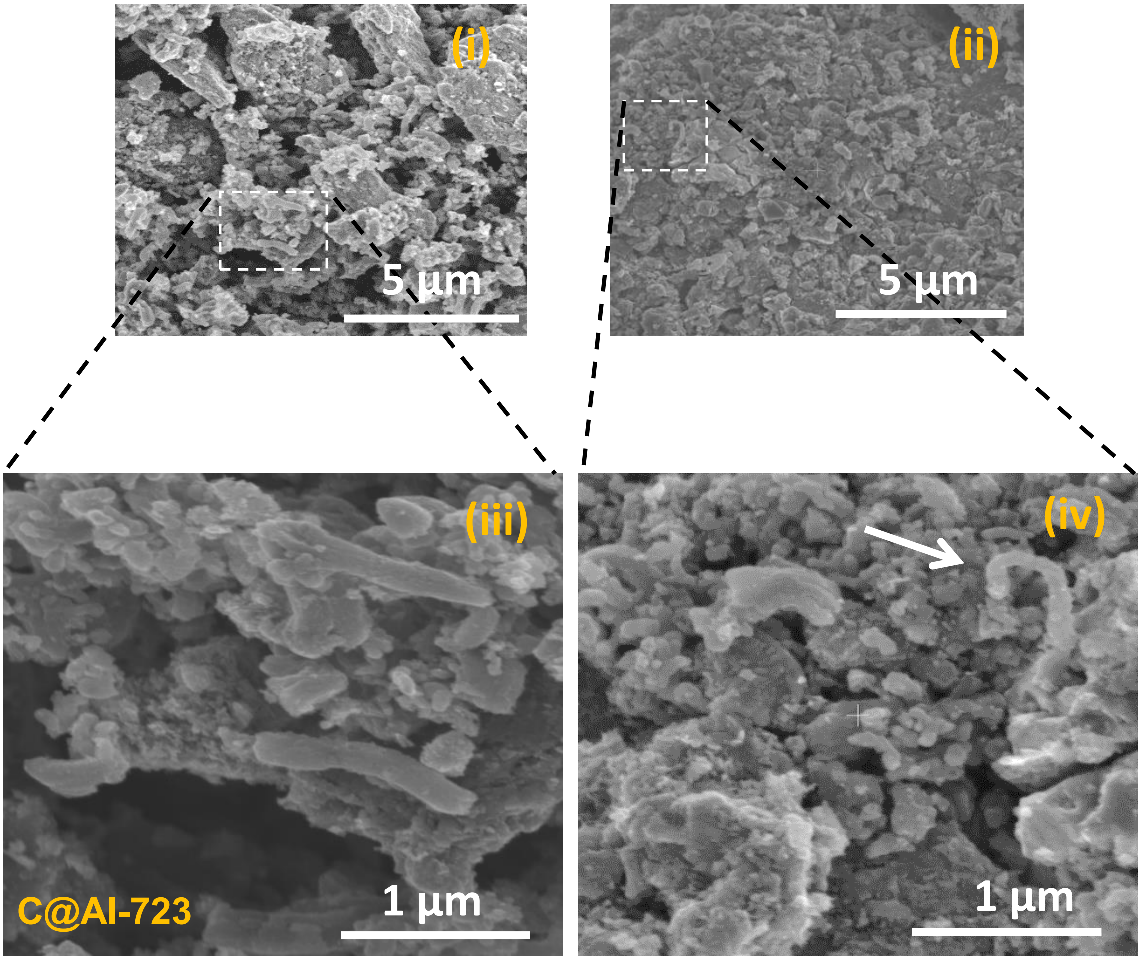

Figure S2. SEM images of C@Al-723 hybrid sample.

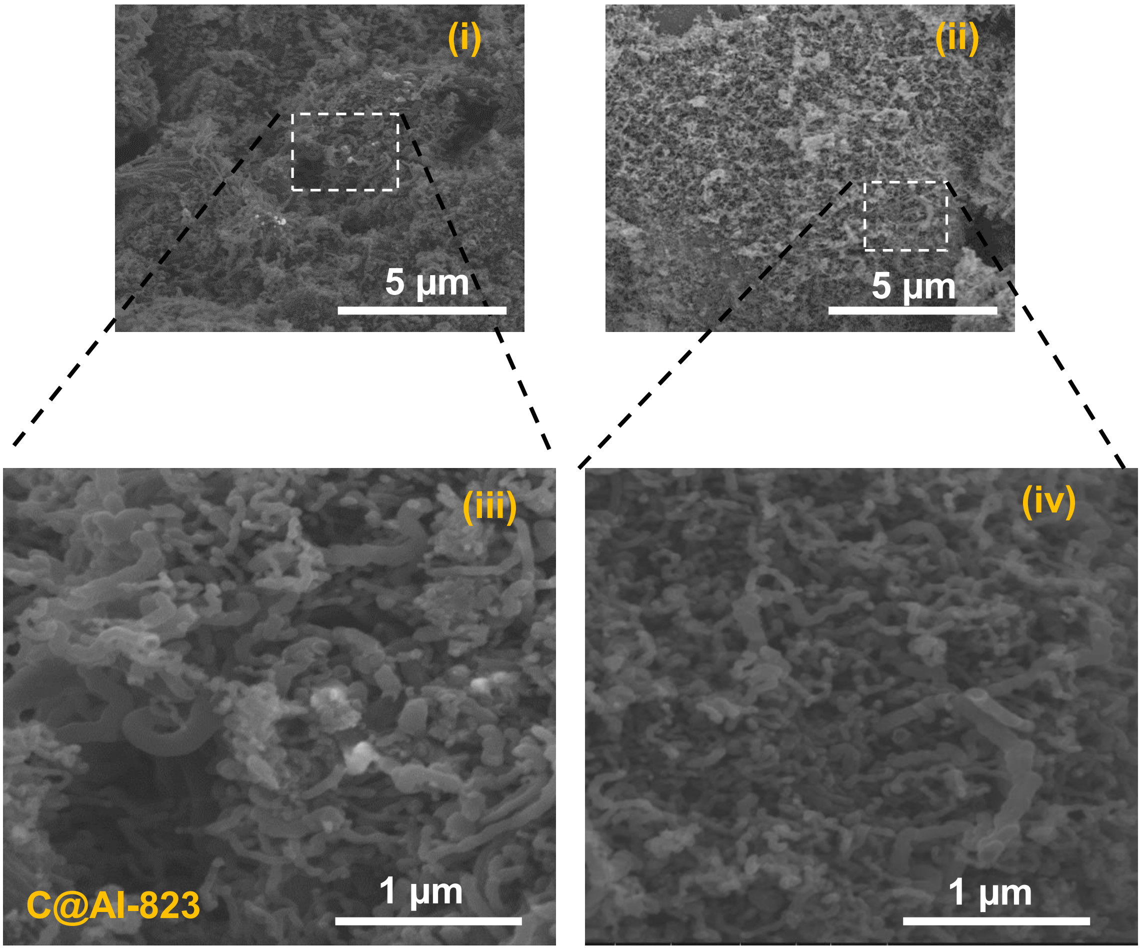

Figure S3. SEM images of C@Al-823 hybrid sample.

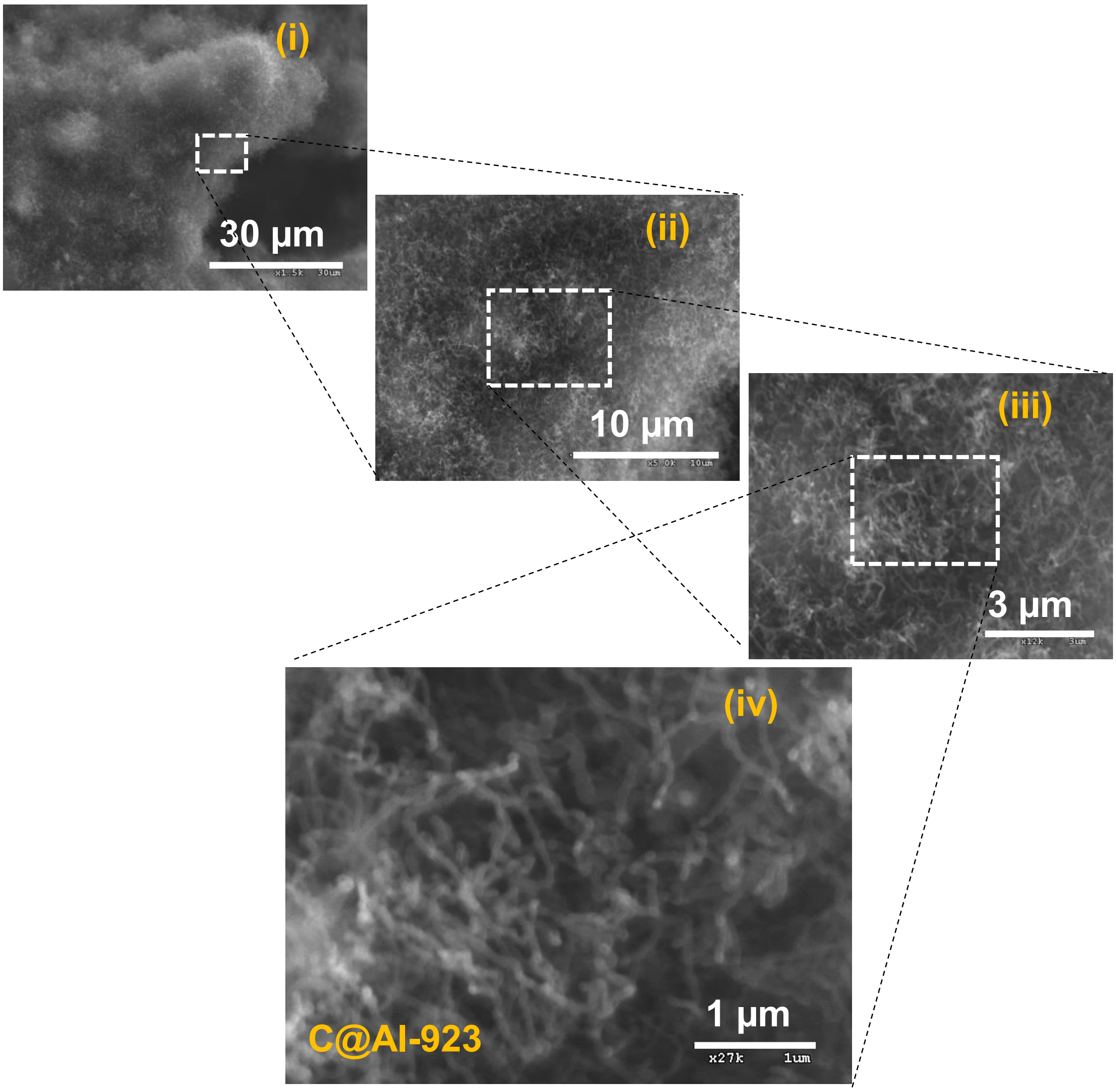

Figure S4. SEM images of C@Al-923 hybrid sample.

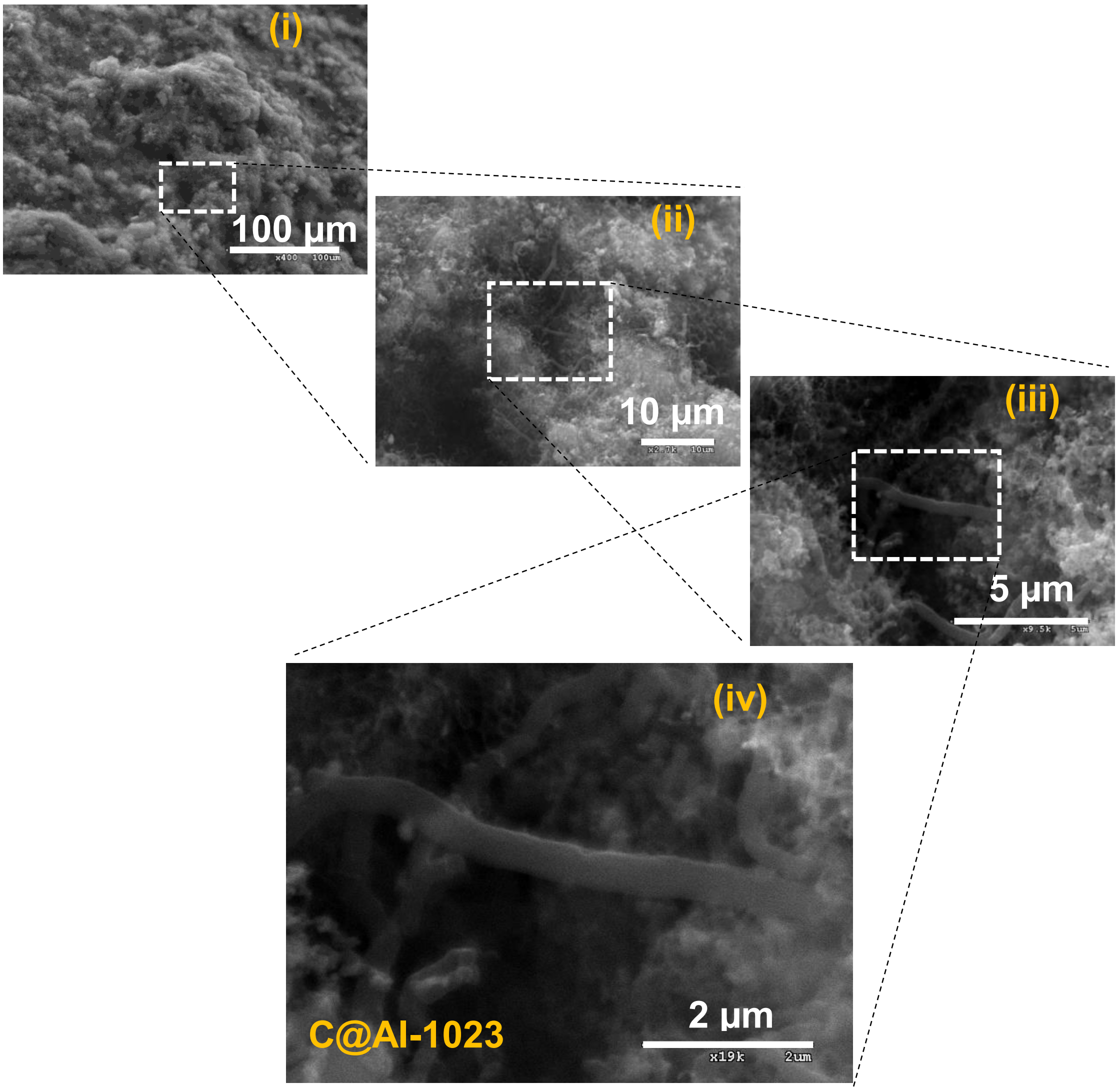

Figure S5. SEM images of C@Al-1023 hybrid sample.

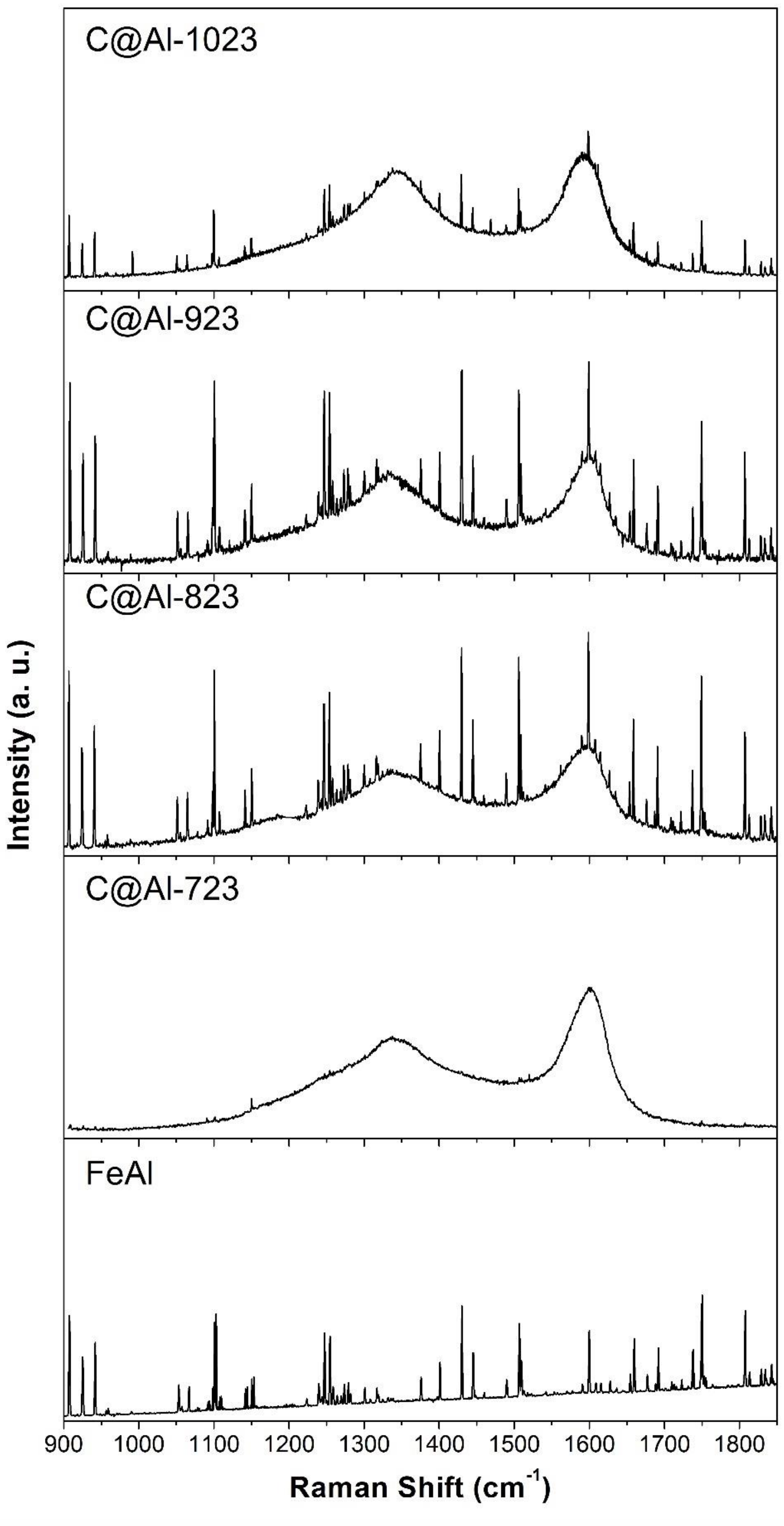

Figure S6. Raman spectra of impregnated sphere (FeAl) and C@Fe-Al_2O_3 hybrids

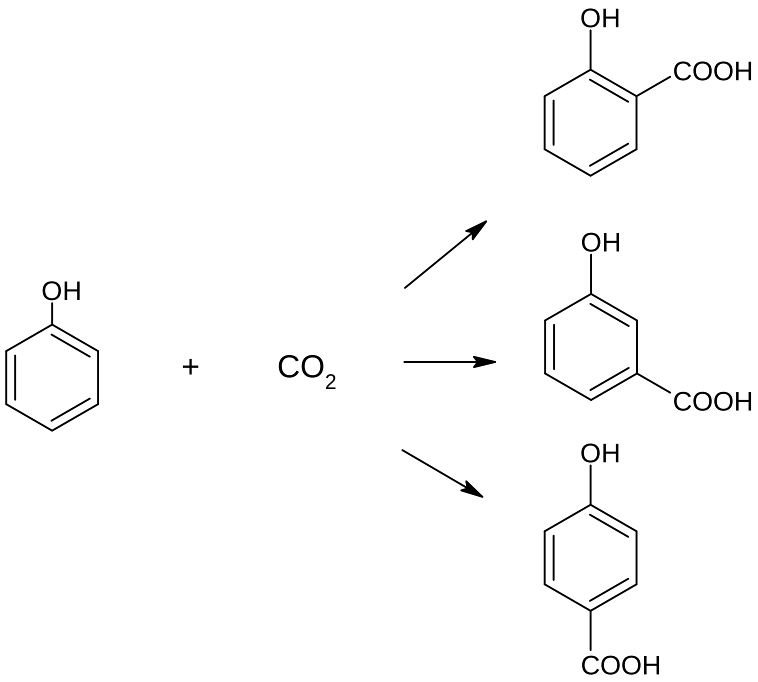

Figure S7. Possible products obtained from direct carboxylation of phenol.
